# Cell-based therapies of autoimmune diseases in the context of artificial intelligence development

**DOI:** 10.1007/s10238-026-02302-4

**Published:** 2026-09-05

**Authors:** Diya Dinesh, Snigdha Suman Das, Barshana Bhattacharya, K. Sreedhara Ranganath Pai

**Affiliations:** Department of Pharmacology, Manipal College of Pharmaceutical Sciences, Manipal College of Pharmaceutical Sciences, Manipal Academy of Higher Education (MAHE), Manipal, 576104 Karnataka India

**Keywords:** Artificial Intelligence (AI), Stem cell research, Autoimmune disease, Immunology, Transcriptomics

## Abstract

Autoimmune diseases are comprised of several chronic inflammatory conditions characterized by activation of autoreactive T and B lymphocytes and produces autoantibodies. While most of the current treatment options mainly rely on non-specific immunosuppression, stem cell based therapies can be a promising candidate for promoting “immune reset” and restore long term tolerance against these complex diseases. Stem cell based therapy eliminate autoreactive clones and reconstitute immune tolerance. Besides, mesenchymal stromal cells (MSCs; also referred to as mesenchymal stem cells) have immunomodulatory effects via paracrine signalling, including anti-inflammatory cytokine and extracellular vesicles secretion. However, successful clinical translation is limited by complex high-dimensional datasets and patient heterogeneity. In this review, we have focused on the convergent journey of Artificial Intelligence (AI) and stem cell based therapies, which mainly evolved in recent times. We also discussed how machine learning and deep learning methods facilitate the analysis of single-cell multi-omics, spatial transcriptomics, and easily identify disease states. Furthermore, the role of AI in optimizing the design and potency of therapeutic modalities, including regulatory T cells (Tregs), Chimeric Antigen Receptor (CAR)-T cells, and mesenchymal stromal cells (MSCs) have also been explored. Altogether, this review highlights the emerging role of AI-guided approaches in bridging mechanistic immunology with future precision stem cell therapies, while several computational and preclinical studies are promising, prospective clinical validation remains necessary before widespread clinical implementation .

## Introduction

Autoimmune diseases arise from loss of immune tolerance, leading to immune mediated destruction of self-tissues, including Systemic Lupus Erythematosus (SLE), Multiple Sclerosis (MS), Rheumatoid Arthritis (RA), Type 1 Diabetes (T1D), Inflammatory Bowel Disease (IBD) and many more. Autoimmune diseases represent a significant and growing global health burden. Although considerable progress has been made in elucidating their pathophysiology, available therapeutic options are still limited, mostly because of the limitations of current treatments like immunosuppressives having major side effects. These immunosuppressive therapies, steroids and biological agents mainly target inflammation pathways [1]. Although these therapies can be effective in managing symptoms, often they do not result in a permanent balance between protective and pathogenic immune responses, which results in severe side effects and inadequate long-term control of disease [2, 3]. The off-target approach of these immunosuppressive strategies increase susceptibility to infections. The need for innovative treatment strategies that minimise the adverse effects while ensuring effective disease management is becoming greater. New strategies such as synthetic immunology are promising alternatives. They rely on designed immune cells such as those that are engineered, synthetic biologics or gene editing to alter immune tolerance and reduce systemic inflammation without impairing overall immune function [2]. New treatment options have already emerged in the form of mesenchymal stem cell (MSC) derived extracellular vehicles (EVs) for better immune modulation. These EVs carry bioactive molecules that modulate key immune pathways and have been shown in preclinical and clinical studies to limit inflammation and promote immune tolerance [4]. Additionally, CAR T-cell therapy has been revolutionary in the field of oncology, and is now being attempted for autoimmune diseases. Genetic engineering of the Tregs are now being introduced to increase their specificity and effectiveness as therapy [5]. The use of Tregs is also a potential candidate because of their significant immunosuppressive properties in controlling autoimmunity. Advances in the field of biotechnology may make it possible to develop personalized therapies using Tregs, to design personalized therapies based on the specific immune profile of the patient [6].

### Pathological framework of autoimmunity

Autoimmune diseases are a heterogeneous group of disorders caused by one or both of the immune systems being capable of recognising and reacting against autoantigens that are normally self-tolerant. The biochemical processes at a cellular level begin with activation of self-reactive T-lymphocytes and then proceed to systematic spread of B-lymphocytes to plasma cells producing destructive autoantibodies. At the same time, the continuous release of pro-inflammatory cytokines leads to a chronic inflammatory environment which eventually results in progressive tissue damage and organ dysfunction. Although there has been improvement in understanding these pathogenic mechanisms, there are no truly novel and specific treatments currently in standard use for this patient population, and treatment is largely based on broad-spectrum systemic immunosuppressive therapy. Although effective at easing symptoms, these methods are nonspecific, have potential for widespread toxicity and make the patient vulnerable to opportunistic infections. Such a widespread limitation in therapy could be viewed as an emergency clinical need to take a cell-based break on precision therapy. The innovative approaches focus on selective rebalancing of immune tolerance and the resulting immune quieting, which should permit remissions lasting for many years, while preserving overall immune competence and efficacy against foreign infections and anti-microbial resistance. Therefore, understanding of the basic mechanism behind autoimmunity continues to inform the development of groundbreaking therapeutic strategies that could restore normal immune function in patients with autoimmunity [7–10].

### The Convergence of cell therapy and artificial intelligence

**Fig. 1:**
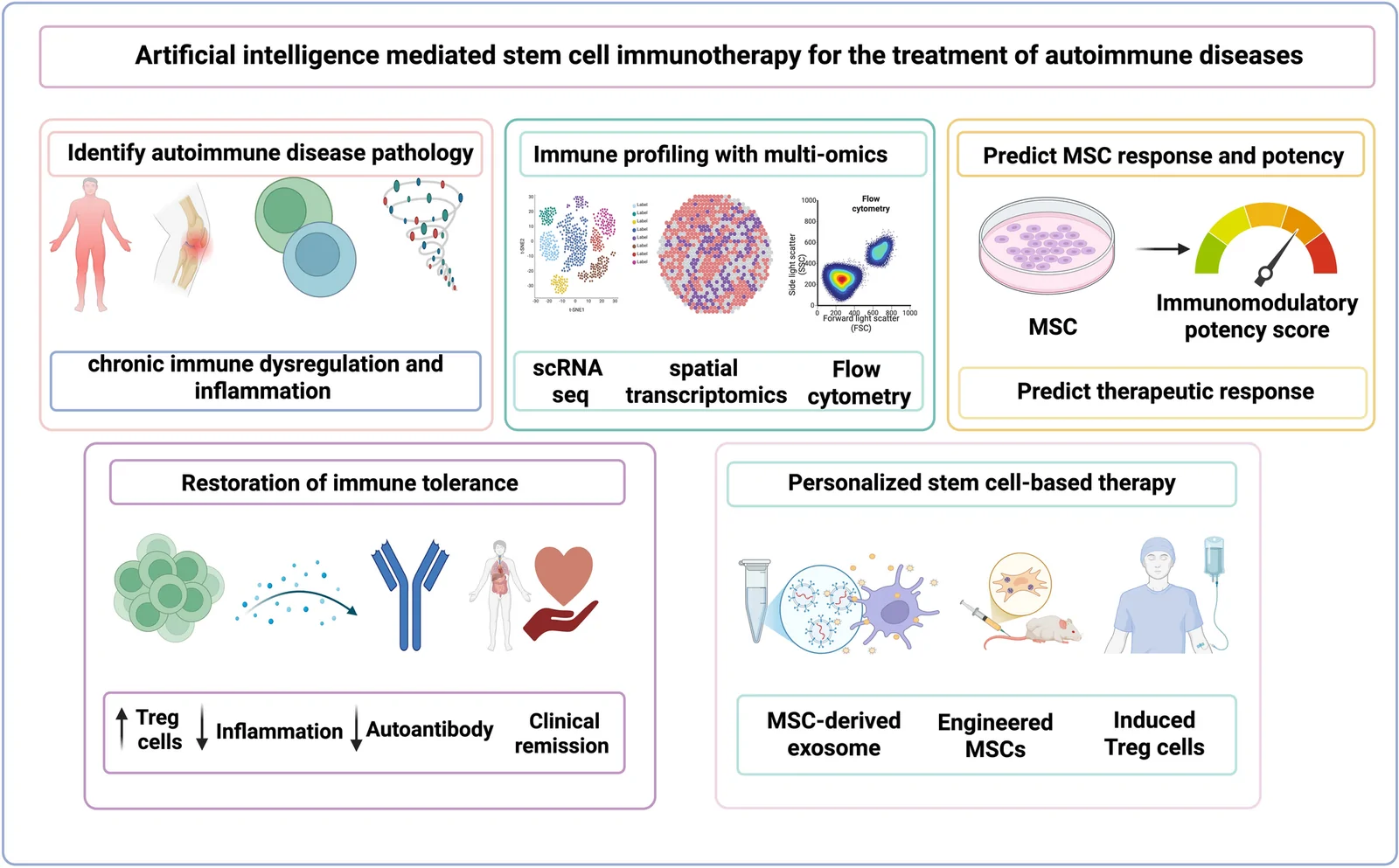
Schematic representation of AI-mediated stem cell immunotherapy for autoimmune diseases. AI-assisted multi-omics profiling (scRNA-seq, spatial transcriptomics, and flow cytometry) identifies disease-specific immune signatures and predicts MSC potency and therapeutic responsiveness. These insights facilitate personalized stem cell-based interventions, including MSC-derived exosomes, engineered MSCs, and induced Treg cells, leading to restoration of immune tolerance, reduced inflammation and autoantibody production, and improved clinical outcomes

The integration of AI with immunology and stem cell-based therapies is essential to combat complex and heterogenous nature of autoimmune diseases. Even though AI-based data analysis and stem cell-mediated research has made their way independently but merging these two emerging sections is still unexplored and need to be unified for better clinical translation. Furthermore, it also examines how AI methodologies, such as machine learning and deep learning help to decode complex immune environment, identify different disease states, and enable predictive and personalized therapeutic strategies (Fig. 1). the role of AI in enhancing the design and optimization of these therapies is also discussed.

Among recent advances, AI serves three core functions in regenerative immunotherapy. First comes its ability to decode complex biological signals - using algorithms trained on vast datasets from single cells, tissue sections, or flow measurements, revealing hidden patterns in immune dysfunction missed by standard methods. Instead of traditional analysis, these models uncover subtle differences among cell types driving disease. Shifting focus, AI has been investigated for improving development pipelines - for instance, refining how stem cell treatments like MSCs, engineered Tregs, or reprogrammed cells are built, tested, and scaled under strict production standards. It estimates effectiveness before clinical use, guiding decisions about which donors or formulations work best. Beyond therapeutic design, another emerging application is the prediction of treatment outcomes using computational models. Here, predictions shape care through tailored patient grouping, flare-up estimation, and adjusting interventions over time based on evolving risk. Structure follows function - the layout mirrors this triad, linking molecular insight to real-world therapy decisions backed by cited evidence [11, 12].

This review focuses at the interface between AI and stem cell-based immunomodulation in autoimmunity with emphasis on mesenchymal stem cells (MSCs; also referred to as mesenchymal stromal cells), Tregs, CAR-T/CAR-Treg constructs and iPS cell-derived products. AI applications to autoimmune diagnostics, imaging analysis and non-cellular pharmacotherapy will be addressed when relevant to patient stratification, biomarker discovery or therapeutic choices regarding stem cell approaches [11, 12].

Moreover, this review systematically interrogates the context of high-resolution cellular datasets in the context of AI, particularly with a focus on single-cell multi-omics and spatial transcriptomics in autoimmune pathologies, to explain cellular heterogeneity and microenvironmental complexities [13, 14]. Failure to include macro-scale clinical informatics, bulk population genetics, and traditional low-throughput screening modalities is warranted because these are lacking in capturing phenotypic drift and spatial architecture that is necessary for advanced cell-based therapies like CAR-T platforms and mesenchymal stromal cell platforms [15, 16]. The ability to compute these high-dimensional single-cell and spatial tracking technologies will ensure a tight connection of the computational tool with a fine immunological scope, without diluting the conceptualisation of the research program with structural cell manufacturing or potency engineering, or with the concept of translatability to barriers [17, 18]. This strategy helps to guarantee the relevance and methodology of new precision immunotherapeutics.

## Data foundations for artificial intelligence in autoimmunity

The broad definition of “AI” which includes structured data resources as well as deep learning. A knowledge base is a well-curated version of data that facilitates most downstream machine-learning analyses, and public data communication repositories including the Gene Expression Omnibus and ClinVar are queryable, curated knowledge bases [19, 20]. These expression resources can also be used with regression-based algorithms along with deconvolution methods like CIBERSORT and RNA-seq reference-based deconvolution, which can recover hidden cell composition information from bulk RNA-seqs with lower cost than single cell approaches [21, 22]. On the other pole, deep-learning approaches like AlphaFold2 can predict the three-dimensional structure of proteins directly from their sequence, and AI-powered tools like PolyPhen-2 provide trained classifiers to forecast the functional impact of missense variants that can be linked to cellular function [23, 24]. Direct application of integrate pipelines that combine protein-protein interaction network analysis, Gene Ontology enrichment, and KEGG pathway analysis has been employed to identify candidate biomarkers and patterns of immune infiltration in these autoimmune diseases such as SLE and the Graves’ disease/Hashimoto’s thyroiditis/psoriasis/T1D panel using workflows that are largely automated [25, 26]. At the end of the day, there is a continuum of LSMs that can also take in and combine ballooning amounts of biomedical literature that are more than just “language polishing” operations.

### Single-cell and multi-omics technologies

The scRNA-seq, cellular indexing of transcriptomes and epitopes by sequencing (CITE-seq), TCR/BCR sequencing, and assay for transposase-accessible chromatin using sequencing (ATAC-seq) play important roles in understanding the spectrum of autoimmune diseases. These high-dimensional technologies collectively generate complex datasets that require integrative computational approaches (Fig. 1).

#### scRNA-seq in autoimmune disease biology

Single-cell RNA sequencing (scRNA-seq) facilitates the identification of rare immune cell populations and their various states, which is essential for comprehending the cellular diversity characteristic of autoimmune diseases. This method enables the identification of novel cell types and states that illustrate the adaptability of the immune system [27]. scRNA-seq offers a detailed view of transcriptomic profiles, which is instrumental in pinpointing potential disease targets and comprehending the mechanisms behind diseases [28]. For Example, the MIRA framework, which combines scRNA-seq and ATAC-seq using multi-modal deep learning. This approach links gene expression with chromatin access, helping pinpoint cell states tied to autoimmune disease genetics [29]. Rather than working in isolation, tools like scANVI (Single-Cell ANNotation using Variational Inference) leverage semi-supervised variational autoencoders to align cell types across diverse autoimmune datasets, boosting accuracy where earlier, fully unsupervised techniques fall short [30](Table 1).

**Table 1:** Role of AI in data interpretation

Types of data	Technique	Clinical & Immunological Application	AI contribution	Primary Computational Challenges	References
Single-cell	scRNA-seq	Identify immune cell heterogeneity	Cell clustering, state detection	Batch effects, high sparsity, dropout events	[27, 28]
	CITE-seq	Protein + transcript integration	Multi-modal learning	Cross-modal feature alignment, scale differences	[31, 32]
	ATAC-seq	Chromatin accessibility	Regulatory network inference	Sparsity, peak alignment across cells	[32, 229]
Spatial	Spatial transcriptomics	Tissue immune niche mapping	Spatial modeling (cell2location, Tangram)	Spatial alignment, cell-type deconvolution pitfalls	[39, 230]
Cytometry	CyTOF	High-dimensional immune profiling	Clustering (FlowSOM, PhenoGraph)	Marker intensity normalization, manual gating bias	[42, 44]
Bulk Multi-Omics	Bulk RNA-seq, Proteomics, Metabolomics	Mapping baseline signaling pathways; identifying diagnostic biomarkers	Network biology, deep biomarker screening	Cell population deconvolution; loss of cellular resolution	[231]
Macro-Scale Clinical	Electronic Health Records (EHR) & Trial Data	Predicting patient outcomes; tracking toxicities (e.g., CRS)	Longitudinal modeling, predictive risk scoring	Handling missing clinical data; standardizing multi-center parameters	[232]

#### CITE-seq’s contribution

CITE-seq builds upon scRNA-seq by simultaneously studying transcriptomes and the protein surfaces, which is critical in defining immune cell phenotypes [31].The technique helps in the determination of the immune cell states between various lineages and tissues through the combination of multimodal data [32].This multimodal analysis is particularly useful in the complex diseases where transcriptional profiles fail to provide the distinguishing information on the immune cells.

#### ATAC-seq insights

Assay for Transposase-Accessible Chromatin using sequencing (ATAC-seq) has shown to be a key epigenetic modality for questioning genome-wide regulatory landscapes and has become an established method for measuring chromatin accessibility. Built upon solid bulk tissue protocols, ATAC-seq can thus be used to achieve high resolution of open chromatin regions that identify active regulatory elements by means of foundational method studies and benchmarked protocols [33, 34]. This ability has been further advanced by the progression to single-cell ATAC-seq (scATAC-seq) technologies, enabling the dissection of chromatin accessibility at cellular resolution and granular insights into cell type–specific gene regulatory programs [33, 35, 36]. These foundational studies primarily propose the molecules and computations programs for chromatin profiling and not autoimmune disorders, but provide the technical and multidisciplinary framework that is important for de-coding cellular heterogeneity in complex tissues. Translationally, open chromatin mapping and characterization of binding sites for transcription factors help identify cryptic non-coding genetic variants and elucidate dysregulated gene regulatory networks that lead to altered, self-reactive immune phenotypes and provide key insight into an epigenome’s role in autoimmunity. In the context of advancing knowledge in immune-mediated pathologies and developing targeted therapeutic regimens, this mechanistic relationship highlights the critical importance of ATAC sequencing–based strategies.

### Artificial intelligence models in extracting disease-relevant cell states

Machine learning and deep learning are the two AI approaches that are essential in processing and extracting meaningful insights out of the large quantities of data produced by these sequencing technologies. The use of AI models also allow predicting disease-relevant cell states using chromatin accessibility and gene expression data (Fig. 1) [29]. They enhance precision in cell type labelling and help to discover genetic differences, which could lead to autoimmune pathogenesis [37].The implementation of AI in the analysis of sequencing data may facilitate identification of regulatory factors and potential biomarkers [11, 12]. Overall, the maturation of single-cell sequencing technology coupled with AI creates a solid foundation of the initiation of the complex biology of autoimmune diseases and preconditions the development of new treatment approaches.

### Spatial transcriptomics & imaging

The use of spatial transcriptomics and multiplex imaging has revolutionized our understanding of immune niches in a variety of tissues (synovium, the pancreas, central nervous system (CNS), and the gut). These technologies determine the spatial organization and activity of immune cells in tissue microenvironments by integrating data regarding gene expression and interactions between proteins. Spatial transcriptomics enables mapping of gene expression in entire tissue samples and retains the spatial context that is required to comprehend cellular interactions and action of the microenvironment. This technique has played a critical role in recognizing distinct gene patterns of expression in immune niches, which are crucial in such tissues as the synovium, pancreas, CNS and gut, where there are specialized immune responses and cellular interactions that control physiology of pathology [38, 39].

On the other hand, multiplex imaging can be used to observe several different proteins on tissue sections simultaneously. It is the method that can strengthen the understanding of cellular composition, cell structure, and their relationships in immune niches and provides spatial and phenotype information on the different cell types. It is particularly useful in immuno-oncology with the focus on the investigation of tumor immune microenvironment as well as the description of cellular response to therapy [40, 41]. Analysis of these datasets require advanced computational approaches. Each of the methods relies on AI, including cell2location, Tangram, and SpaGCN, which helps analyse and combine spatial transcriptomics and multiplex imaging data to gain a better insight into tissue-specific immune niches. Spatial transcriptomics combined with multiplex imaging and AI tools can help a researcher better understand immune niches within complicated tissues. These studies help to understand how immune cells interact with their micro-environment which may serve to provide therapeutic treatments to various diseases such as cancer, autoimmune and infections.

### High-dimensional cytometry

CyTOF (cytometry by time-of-flight) and imaging flow cytometry have the capability to perform high-dimensional immune profiling, which might give pertinent information on cell characteristics and functions on a single-cell level. CyTOF employs a mass spectrometry technique that labels antibodies with metal isotopes giving the capability to measure many markers simultaneously without the spectral overlapping problems that flow cytometry can cause. The capability enables a detailed analysis of complicated biospecimens that contain a great number of cellular markers, and this tool is indispensable in the cellular and proteomic study [42–44]. Imaging flow cytometry is a combination of the high-throughput capabilities of flow cytometry and the high spatial resolution of fluorescence microscopy. Current developments can now provide high-resolution three-dimensional cell imaging, with cellular structures down to the level of subcellular structures visible, and can further enhance the study of complex cellular interactions during immune responses [45, 46]. With these developments, it is now possible to do very specific characterizations of immune cell subsets, states of functional behaviour, and their interactions. The algorithms such as FlowSOM and PhenoGraph are clustering algorithms, important in the analysis of the high-dimensional data generated by these technologies. FlowSOM is based on self-organizing maps as its method of data structuring to help in efficient visualization and cell populations identification, whereas, PhenoGraph uses graph-based clustering, which is especially useful to process large single-cell datasets to generate significant biological lessons, including cell type specific interactions and rare cell groups.

These algorithms enhance the capacity in detecting rare subsets of immune cells associated with tolerance that can be invaluable as a source of understanding and influencing immune responses potentially with therapeutic benefit. The tools of AI, such as machine learning models like DeepCyTOF and DeepScana introduce advanced codes to process information and identify cells, providing the astonishing level of accuracy and efficiency. Such methods also automate and improve the analysis of large datasets as well as help identify and characterize rare cell populations in diverse biological samples, contributing to the understanding of the dynamics of the immune system and using therapeutic targets [47–49]. Altogether, the combination of high-dimensional profiling technologies and AI-based clustering algorithms is an important breakthrough in immunology research that would allow identifying the new immune subsets and the functions they play in health and disease (Fig. 1).

### Data challenges

Advanced therapeutics have the potential to leverage AI frameworks by harnessing a wide range of machine learning algorithms to uncover valuable insights from the intricate biomedical data. The first step to systematically examine the practical difficulties of deployment, data non-uniformity and constraints on interpretability is to define the operating modes of such underlying computational architectures. For example, random forest and support vector machine algorithms are supervised learning algorithms that use historical, expert-labeled data sets to identify predictive patterns. They can be used to map inputs to known results for either classification or regression, thus making them highly useful for diagnosis, prediction of prognosis and patient stratification for disease prediction, based on multi-omic biomarker profiles. In the case of unstructured and high dimensional data like raw medical images or signals over time, deep learning architectures (and artificial neural networks, in particular, convolutional neural networks (CNNs) concatenate multiple layers of interconnected nodes that automatically extract, and hierarchically combine, visual features without requiring manual feature engineering, thereby outperforming existing methods in radiology diagnostics and advanced clinical pattern recognition.Unlike these predictive models, unsupervised clustering methods consider completely unlabeled data, automatically defining clusters, latent patterns or groups of data according to just a mathematical similarity. Examples are k-means clustering and t-distributed Stochastic Neighbor Embedding (t-SNE). This is an essential tool for exploratory data analysis, cell phenotyping and curation of unique patient sub-groups needed to support personalized medicine strategies. Finally, for dynamic operational environments, reinforcement learning algorithms optimize sequential clinical decision making by stepping through an iterative sequence of trial, error, and dynamic feedback and instructing algorithms and computation methods based on the dynamic feedback from the patient to maximize the defined therapeutic rewards. Last but not least, the different computational paradigms shed light on the specific algorithmic challenges associated with clinical translation. They directly support the systemic challenges, including the transparency of models, the explainability of “black-box” model, the incorporation of highly heterogeneous clinical data in the multimodal dimension, as well as the dynamic time dimension of treatment, which needs to be solved to create credible, interpretable, and clinically viable AI tools for healthcare [50–53].

#### Small patient populations

Autoimmune diseases typically possess a small sample rate, meaning that research data comprises small population samples. AI is able to overcome this difficulty through data augmentation and transfer learning methods. Transfer learning allows the models that were trained on larger and related datasets to be applied to smaller datasets and increase their generalisation capacity [54, 55].

#### Batch effects

Batch effects are those that occur due to data collected in different settings and environments, i.e. different laboratories, equipment etc., which can bring about inconsistencies which do not pertain to the biological variables under study. To do that, the batch effect correction algorithm (BECA) and AI-inspired approaches can be used to identify and correct these errors thus improving the validity of the analyses [56].

#### Heterogeneity

There is high heterogeneity in autoimmune diseases as they have different clinical manifestations and a wide range of pathophysiological processes. To comprehend this intricacy, AI techniques, in particular, deep learning, can analyze multidimensional complex data provided by genomic data, phenotypic data, and imaging data. Through machine learning model training one can establish subgroups in heterogeneous data enabling more specific ways of treating these groups [11, 12].

#### Class imbalance

This occurs when one of the classes is underrepresented, e.g. a particular outcome, subtype of disease. The AI can solve class imbalance through resampling techniques, such as oversampling the minority and under sampling the majority classes, and also ensemble techniques. These are some of the ways of creating balanced datasets that will improve the ability of the classifier to make accurate predictions [57–59]. AI is still developing and offers more effective tools to solve such problems in autoimmune datasets, which enhances the chance of making valid and significant scientific decisions. Overall, the integration of multi-omics datasets with AI forms a robust framework for decoding autoimmune disease mechanisms (Fig. 1).

Through AI-driven omics methods, harmful immune cell forms and their control systems come into view - shaping how antigens are chosen, effectiveness refined, individuals grouped for treatment in the stem cell therapy framework laid out in Sect.  3 [11, 29].

## Advanced cell-based therapeutic modalities and computational frameworks enhanced by artificial intelligence

Section 3 acknowledges that today’s advanced therapeutic options for autoimmune disorders are a wide spectrum of emerging technologies that span the fields of biology and engineering. This spectrum includes adaptive cellular platforms that are more mature, including conventional CAR-T cells and regulatory CAR-Tregs that are engineered to modulate the immune system. At the same time, strategies involving adult and pluripotent stem cells, such as MSCs, have regenerative and immunomodulatory properties. In addition to these experimental platforms are advanced in silico computational forecasting platforms, including digital twins, which can be used to predict disease processes and treatment effects in individual patients. AI serves as the linkage and optimisation hub that brings all these architectural components together, and helps to improve design, implementation, and prediction of the therapeutic and computational processes, thereby enhancing precision and efficacy in managing autoimmune diseases [60–63].

### Adoptive T-Cell architectures: conventional CAR-T and CAR-Tregs

Tregs and CAR-Tregs have great potential as treatments for autoimmune disorders, contributing to their role in maintaining the immune tolerance and inhibiting autoimmunity through their intrinsic role. The recent advances in CAR-T and CAR-Treg treatments have shown satisfying results in efficacy following the identification of specific antigen targets and alteration of cellular response. CAR-T cell therapy is most successful at combating hematological cancers, with several ART treatments approved by the FDA as well as clinical trial studies involving these methods [64]. The CAR-based strategies development on Tregs aims to leverage on these immunosuppressive properties for wider use in Tregs for therapeutic in autoimmunity [65]. AI plays an important role in a number of critical aspects of CAR-Treg development, including antigen selection, CAR design, off-target prediction, and dosing. AI techniques such as deep learning and reinforcement learning are used for developing CAR constructs that are capable of recognizing diverse antigen profiles and thus reducing the issue of antigen escape and increasing efficacy [66]. AI has shown promise in predicting off-target effects by analysing large datasets and identifying the potential non-target interaction of the therapy, helping to improve the safety profile of these therapies. In the context of CAR-Treg dosing, reinforcement learning frameworks have been modeled to simulate patient-specific immune dynamics and optimize infusion schedules that minimize systemic immunosuppression while maintaining antigen-specific tolerance, though these remain preclinical computational studies requiring prospective clinical validation [66, 67].

However, there are still some problems in terms of widespread introduction of Treg and CAR-Treg therapies in clinical settings. Major hurdles are ensuring durability of therapeutic response, precise tissue targeting of therapeutic cells, safety considerations e.g. potential oncogenesis and immunosuppression on systemic level [66, 68]. In particular the durability of response is difficult with potential activation induced cell death or loss of CAR expression requiring strategies for prolonged functions of therapeutic cells [69]. Additionally, getting these cells to sites of disease remains a great challenge if they are to be accurately directed (Table 2).

**Table 2:** AI applications across advanced cellular therapies and predictive bio-modeling

Therapeutic platform	Biological Role	AI Method/Tool	Specific Example/Model	Clinical Validation Stage	References
Tregs / CAR-Tregs	Immune suppression; antigen-specific tolerance	Deep learning, reinforcement learning	STAPLER (TCR-peptide specificity); RL-based dosing simulation	Preclinical / computational	[66, 161, 233]
CAR-T (CD19)	B-cell depletion; immune reset	ML for CRS prediction; patient stratification	Pre-infusion cytokine ML model (AUC > 0.80 for grade ≥ 3 CRS)	Phase I/II case series	[72, 74, 85, 234, 235]
MSCs	Immunomodulation; paracrine signalling	CNN / R-CNN; random forest; secretome ML	Cascade R-CNN for senescence detection; RF for donor potency stratification	GMP/preclinical; ongoing trials	[116–118, 120, 236]
iPSCs	Cell replacement; beta-cell generation	AI-guided differentiation; clone QC	ML-based iPSC clone quality classifier; image-based differentiation staging	Preclinical / in vitro	[139, 140, 162–164, 237]
HSCs	Immune reconstitution; long-term remission	Risk prediction models	AI-based engraftment risk scoring; tumorigenicity detection	Preclinical	[142, 238]
Digital Twins	Patient-specific in silico simulation	ODE-based AI models	Mathematical immune response models for autoimmunity	Theoretical / in silico only; no RCT validation	[143, 144]

AI-driven strategies can be offered to overcome these translational challenges successfully. The creation of digital twins - virtual models of patients that can mimic biological processes - has the potential to support the concept of personalized medicine and predict unique treatment outcomes. Such models enable the possibility to predict long-term efficacy and side effects, which has the potential to inform a clinical decision-making process [70]. Furthermore, combining the data from multi-omics, such as genomics, transcriptomics, and proteomics, can yield a complete picture of the disease landscape of a given patient. This integration facilitates the development of precision therapeutics, at a personalized molecular profile, which may improve the target to tissues and reduce the off-target consequence [11]. Overall, the development of Treg and CAR-Treg therapeutics for autoimmunity requires addressing substantial translated barriers, as well as maximizing utilization of AI to optimize and improve therapeutic design, administration and efficacy (Fig. 1).

#### Translational barriers in CAR-T and CAR-Treg therapy for autoimmunity

In this context, CAR-T and CAR-Treg therapy showed high potential in autoimmune disease, and several translational limitations should be overcome before becoming broadly available in the clinic. One of the most important challenges is the exhaustion of T cells: persistent antigen exposure from the inflammatory auto-immune environment causes steady increase of inhibitory receptors, namely PD-1, LAG-3 and TIM-3, that limits the persistence of CAR-T cells over time [71, 72]. Longitudinal scRNA-seq studies of CAR-T persistence are currently used to develop AI-based transcriptomic classifiers, to identify exhausted TCR populations in CD45RO + and CD45RO- T cells, allowing for the selection of starting material with greater longterm functional potential [66, 73].

While not as severe as with oncology, cytokine release syndrome (CRS) is a clinically significant safety issue with autoimmune indications. Machine learning models trained on pre-infusion biomarker panel measurements (serum ferritin, IL-6, and C-reactive protein) have been shown to have AUC values > 0.8 for retrospective prediction of grade ≥ 2 CRS, providing a pathway for infusion risk stratification [74]. Two major challenges still exist: trafficking of tissues and homing: systemically infused CAR-Tregs must find their way through inflammatory endothelium, compete with effector lymphocytes to adhere and infiltrate, and tolerate the pro-inflammatory environment that was salient for their effector function [68, 75]. A spatial transcriptomics analysis of the chemotactic environment of the tumour in targeted regions provides a new way to relate batches of candidate CAR-Tregs to their expected tissue distribution and a computational strategy to optimize the engineering of their homing receptors before clinical infusion [76].

Clinical scalability is further restricted, due to the complexity of the manufacturing process. Manufacturing of autologous CAR-Treg products involves multiple testing steps (e.g., quality controls) that typically take 2–4 weeks (which may not be suitable for rapidly deteriorating patients) in closed-system GMP production [77]. Currently, automated bioreactor platforms utilize AI-enabled process analytical technology (PAT) to actively track, measure, and report cell viability, cell expansion, and cell surface phenotype parameters on an in-line basis, and trigger automated process corrections upon deviation from user-set parameters and quality thresholds [78, 79]. Lastly, because CAR expression may be lost over time due to silencing of the endogenous gene or destruction of CAR+ cells in vivo by activation, longitudinal monitoring strategies are necessary; electronic health record-based digital health platforms with AI algorithms that incorporate trends in biomarker measurements may facilitate early detection of functional relapse [72, 80].

### Conventional CAR-T in autoimmunity

CAR-T cell therapy is an adoptive immunotherapy strategy in which autologous T-cells are genetically engineered with synthetic receptors that confer specificity against defined antigens. The CAR structure is composed of an extracellular single-chain variable fragment (scFv) from monoclonal antibodies that binds to a specific ligand, attached via a transmembrane domain to a set of intracellular signalling modules—typically incorporating costimulatory domains like 4-1BB or CD28 alongside a primary CD3-zeta signaling domain. This design allows these engineered T cells to mediate effector functions in an MHC-unrestricted manner, facilitating direct binding of target antigens without traditional peptide presentation by the MHC. In autoimmune diseases, CAR T cells have been effectively redirected to target B-cell lineage–specific surface antigens, such as CD19, enabling profound and selective depletion of pathogenic, autoantibody-producing B-cell clones. This targeted cytotoxic deletion creates a “cellular reset,” eliminating aberrant immune effectors and facilitating the restoration of durable immune tolerance. Thus, CAR T-based therapy emerges as a transformative modality capable of achieving prolonged, drug-free clinical responses, in stark contrast to traditional immunosuppressive or antibody-based B-cell depletion approaches [81–84].

CD19 CAR-T cells, which was first developed to help patients with B-cell cancers, are now being started to be used for treating autoimmune diseases, such as SLE and myositis. This transition is inspired by the findings that targeting B cells containing a protein called CD19, can help alter certain immune responses in an effective way, which may help curb the symptoms of an autoimmune disease.CD19 targeting CAR-T cells have achieved promising results in autoimmune rheumatic diseases with rapid and significant therapeutic benefits over existing therapies. This technique is already accessible to drug-free remissions and manageable side effects in such diseases as SLE and other autoimmune diseases [85]. These positive results are causing other studies to focus on broad, long-term ones to verify the safety, effectiveness and permanence of CAR T therapy in autoimmune spaces. In short AI, is playing a role that is becoming very critical in the improvement and expansion of CAR-T cell therapy [72]. AI plays a huge role in different fields, such as patient classification, toxicity prediction, or relapse monitoring. AI technologies are under development to predict the success and side effects of CAR-T therapies with the help of machine learning algorithms involving complex biological data [66]. These tools help to identify patient groups most likely to benefit from CAR-T therapy whilst reducing the adverse effects. In addition, AI may support clinical decision-making such as analysing patient-reported outcomes, assisting in the monitoring and management of possible toxicities [80]. Predictive biomarkers, enhanced by AI, play an important role in anticipating the response of the therapy and the cases where potency might return. They help to balance the effectiveness and toxicity as the insights gained about the dynamics of CAR-T cell persistence and associated dangers such as cytokines release syndrome [74]. Machine learning models trained on pre-infusion cytokine profiles including IL-6, ferritin, and C-reactive protein levels have shown predictive value for identifying patients at high risk of grade ≥ 3 CRS following CAR-T infusion, have demonstrated retrospective predictive accuracy for grade ≥ 2 CRS [87]. These models, originally developed in oncology settings, are now being evaluated for their applicability in autoimmune CAR-T trials [72]. These advancements indicate that when CAR-T therapy is integrated with AI, it may help develop a more personalized approach to treatment, thereby leading to more accurate and targeted strategies, ultimately improving the patient’s outcome in both cancerous and autoimmune diseases [72, 80].

### Mesenchymal and pluripotent stem cell paradigms (MSCs/iPSCs)

The complexity of the challenges in moving from bench to bedside in stem cell-based therapeutics for autoimmune diseases necessitates the establishment of foundational biological characteristics of both MSCs and induced pluripotent stem cells (iPSCs) before tackling the next step of developing human therapies. MSCs possess immunomodulatory secretomes and low immunogenic profiles, enabling them to exert immune regulation in inflammatory settings, and have been obtained from various sources like bone marrow, adipose tissue, and umbilical cord matrix [88, 89]. In contrast, engineering by transcription factors (ETs) can convert mature somatic cells to become iPSCs, which are cells with a morphology similar to that of embryonic cells, have the capacity to self-renew indefinitely, and are broadly pluripotent [90, 91]. Although iPSCs hold great promise for clinical translation, the biological foundation is set when considering the immunomodulatory properties of MSCs and their virtually limitless ability to proliferate and differentiate, and how these will overcome subsequent manufacturing hurdles, standardize their nomenclature, and mitigate clinical translation challenges.

Throughout the review, the term “mesenchymal stem cells (MSCs)” has been used, but it has been noted that no universal consensus exists about the rejection of “stem cell” to “mesenchymal stromal cell” as the term is only relevant for subpopulations with the most stringent self-renewal and multipotency requirements. Likewise, the term “medicinal signalling cells” suggests they aren’t important for direct replacement; they’re rather for producing trophic and immunomodulatory factors. Despite the agreement reached by the International Society for Cell & Gene Therapy (ISCT), however; standard cultures are highly heterogeneous as true mesenchymal stem cells are found in very small numbers, around 1 in 10,000–100,000 of the cells in a bulk sample [92–94]. In addition to immune privilege, MSCs mediate some of the effects of their immunotherapy by actively migrating and homing to sites of tissue injury and active inflammation. As soon as they arrive at the MSC niche, they sense the local inflammatory cues and actively recruit and re-educate multiple immune cell subtypes, providing the scientific underpinning for their therapeutic value [95].

The scientific characterization of MSC in immune function has increased significantly. Their wide-ranging inhibitory effects such as modulation through toll-like receptor activation and direct interactions with T cells, NK cells and dendritic cells prompted the designation of their inhibitory functions as immune system agents [96, 97]. Subsequent reports of antibody generation against, and rejection of, allogeneic donor MSCs in some settings complicated this simple “immune privilege” framing, prompting a reconceptualisation of MSCs as “immune evasive” rather than truly immune privileged [98, 99]. More recent studies have extended this scrutiny to specific quiescent subpopulations, showing that nestin-GFP+ bone marrow progenitors, for example, have a strong ability to evade immune surveillance after transplantation in a host previously immune to the donor antigen [100, 101], while quiescent stem cell populations more broadly are shown to evade immune surveillance through mechanisms separate from their proliferating descendants [102, 103]. In addition, mesenchymal-derived multipotent MUSE cells have been designated as having a unique immune-privilege phenotype which is translationally relevant for inflammatory disease [104, 105]. Combined, these findings suggest that immune privilege in the mesenchymal compartment exists in subsetual local populations but not in bulk preparations of MSCs, with important consequences for potency and quality control models designed on the basis of artificial immunity, which would require an approach to MSC immune profile instead of a homogeneous product [106].

The therapeutic implementation of MSCs in autoimmune indications relies on core physiological attributes beyond simple immune privilege, characterized primarily by active tissue homing and dynamic microenvironmental immune-reprogramming. Systemically infused MSCs utilise an active migration paradigm to home directly toward chemical distress gradients generated at sites of active tissue injury, progressive cellular hypoxia, and acute inflammation [107–110]. Upon infiltration, MSCs do not act as passive entities; rather, they serve as integrated sensor units within their immediate cellular niche, initiating robust immunoregulatory signalling pathways through toll-like receptor (TLR) modulation and direct cellular cross-talk [96, 97]. This microenvironmental system actively responds to localised pro-inflammatory signals, specifically tumour necrosis factor-alpha (TNF-α) and interferon-gamma (IFN-ᵞ) to physically recruit and systematically reprogram highly diverse host immune populations [111–113]. By modulating effector lymphocyte activation, suppressing autoreactive clones, and shifting helper populations toward an anti-inflammatory, homeostatic phenotype, MSCs act as a vital cellular cornerstone for establishing long-term immune tolerance in complex immune-mediated pathologies [114, 115].

AI improves the prediction accuracy of mesenchymal stem cell (MSC) potency. Specifically, a morphology-based cascade R-CNN deep learning model achieved high accuracy in detecting senescent MSCs from brightfield microscopy images alone, enabling non-invasive quality screening during GMP expansion without requiring senescence-associated beta-galactosidase staining [116]. Furthermore, machine learning classifiers trained on integrated transcriptomic and secretome profiles have been shown to discriminate between high-potency and low-potency MSC donors prior to large-scale expansion, with donor sex, passage number, and cytokine secretion profiles identified as key predictive features [117, 118]. Imaging data is also a part of the assessment of MSCs as it provides visual information about cellular behaviour and phenotypic expression necessary for predicting potency and therapeutic viability. AI’s processing capability of complex image information enables complex analysis and interpretation on complex and detailed MSC characterization [119]. In terms of donor selection, AI determines genetic and phenotypic disparities between prospective donors, and factors include MSCs doubling time, MSC proliferation rates, and gene expression studies. These criteria allow the AI models to assess variability in the donors and foresee the bone-forming potential of the MSCs, therefore, helping to select the best donors for therapeutic use [117, 118]. AI systems can also identify variations in the established protocols for production to ensure high-quality production that meets regulatory standards, ensuring therapeutic effectiveness and safety of MSC-based products [120].

#### Translational barriers and artificial intelligence-driven mitigation strategies

MSCs have a variety of biological activities essential to their therapeutic potential. The functions are central with secretion of various immunomodulatory molecules that have a capacity to regulate immune responses by supporting anti-inflammatory phenotypes and bringing down the exaggerated immune activation. MSCs dynamically interact with Toll-like receptors (TLRs) that are well-known sensors of exogenous pathogens, and endogenous danger signals; TLR activation switches MSC phenotype from either pro-inflammatory or anti-inflammatory, subsequently affecting their immunomodulatory properties and interactions with innate and adaptive immune cells [121–123]. By these pathways, MSCs have a strong impact on the inflammatory landscape of the microenvironment, affecting the cytokine milieu and the polarity of immune cells such as the induction of M2 macrophages and increased regulatory T cell populations, all of which contribute to tissue repair and immune homeostasis [124].

Although MSCs have demonstrated immunomodulatory activity, it has been difficult to apply them clinically due to a number of unanswered questions. A major challenge is donor heterogeneity, because MSCs from various donors have different immunosuppressive ability, growth and production of cytokines, which causes variable clinical results in different studies [117, 118]. The manufacturing inconsistency exacerbates this issue, as the inter-batch variability that is caused by differences in culture conditions, different passage numbers, and different bioreactor parameters is hard to detect using traditional release assays [125, 126]. During large-scale expansion, culture-induced senescence, resulting from an increase in oxidative stress from replicative exhaustion, progressively degrades the therapeutic potential of MSCs, characterized by the increase in cell size, reduced markers expressions and differentiation potential [125, 126]. In addition, the balancing act between engraftment in vivo after systemic infusion and trapping in lungs most of the cells upon injection (in large part due to selectin homing to sites of inflammation) severely limits the time of immunomodulatory signaling. Last but not least, a few published randomized controlled trials (RCT) have demonstrated short-term or long-term partial improvements in RA, Crohn’s disease, and SLE patients have been treated with MSC, which necessitates better stratification of patients and selection of more appropriately potent products in order to obtain better results.

However, challenges to that include but are not limited to those which can be addressed by AI approaches, such as Machine Learning (ML) and Deep Learning (DL) algorithms. AI models can be used for predicting optimal MSC behavior and functional phenotypes with high accuracy by incorporating and analyzing the data from high-throughput screening assays (e.g., the morphology captured by single-cell images) that include datasets generated from genomics, transcriptomics, proteomics, metabolomics, and live cell imaging [127–129]. These computational structures will perform essential functional characterizations of cells without harming or requiring sacrifice of the cells, provide a robust solution to cell functional characterization that is objective, scalable, and quick, compared to conventional assays, and will transform the cell functional characterization field. Thus, AI-powered analysis can optimize the clinical translation process by improving standardization, forecasting treatment efficacy and mitigating risks from regulatory and manufacturing which are historically involved in the preparation of advanced therapy medicinal products (ATMPs) using MSCs [130].

In the past few years, AI driven solutions have started to take care of multiple of these barriers at the same time. The use of morphology-based deep learning models that recognize senescent MSCs from images acquired with non-invasive, brightfield imaging to allow for real time quality gating without any assays of MSCs consumed during this process [116]. Secretome profiling combined with machine learning also allows for the screening of prospective donors using a molecular signatory and EV miRNA signature to predict immunosuppressive potency before committing to the donor [131, 132]. Closed bioreactor systems integrated with AI-powered process analytical technology (PAT) allow companies to automatically monitor key quality parameters online while also identifying deviations from specific process window [78, 79]. This not only enhances manufacturing consistency, but also supports optimization. To develop a machine learning model for patient stratification, multi-omics signatures are developing that will predict the response to the treatment provided by the MSCs with immunosuppressive activity and will allow for precision matching between patient immune phenotype and the specific MSC product used for treatment [11].

Furthermore, classical immunosuppression by cytokines, there are some new mechanisms that the therapeutic role of MSCs is increasingly being discussed. These EVs derived from MSCs contain bioactive molecules and exhibit anti-proliferative effects against autoreactive T cells and pro-polarizing effects on macrophages through their bioactive molecules (miR-21, miR-146a, TGF-β1, and proata-G is prostaglandin E2) without necessitating direct cell-cell contact [4]. The use of AI-assisted multi-parameter characterization of EV cargo by proteomics and small RNA sequencing is rising as an EV potency surrogate to help identify EV batches with better anti-inflammatory properties at their point of manufacture [133]. Mitochondrial transfer to metabolically stressed immune cells (such as T cells or macrophages at inflammatory sites) is another mechanism that MSCs use to repair mitochondrial function and halt inflammatory effector immune responses; the mitochondrial fitness of MSCs before infusion is now evaluated via AI-based-metabolomic profiling to indicate a functional parameter [134]. Differential capacity for indoleamine 2,3-dioxygenase (IDO) expression and T-cell suppression have been associated with reprogramming of metabolic programs in MSC subpopulations as shifting between glycolytic and oxidative phosphorylation states determined by AI clustering at the single-cell level and metabolomics [134, 135]. Additionally, the advent of single cell RNA-sequencing, has shown that important and clinically relevant subsets of MSCs are heterogeneous, including CD146-marked perivascular progenitors and adipogenic-biased subclones, which are distinguished by not being identifiable using traditional surface marker panels [136]. The mark of AI-driven, prospectively isolated defined MSC subsets by single cell phenotypic signatures is the potential to create a standardized immunomodulatory potency of Clinical MSC products.

### iPSCs & HSCs

AI has had a significant role in the promotion of processes having a relationship to induced pluripotent stem cells (iPSCs) and hematopoietic stem cells (HSCs) in several important areas, including reprogramming, prediction of differentiation, clone quality assessment and detection of the risk of tumorigenesis.

Refractory autoimmune diseases could be impacted systemically by induced pluripotent stem cells (iPSCs) and targeted by hematopoietic stem cells (HSCs). HSC transplantation with both autologous and allogeneic sources applies intensive conditioning approaches to remove the patient’s abnormal, self-deceptive immune repertoire, helping to provide a foundation for reconstitution of the immune system and restore self-tolerance. At the same time, using iPSCs as a clone, which is derived from a patient’s own cells or has been originated from a universal donor, provides a renewable “off-the-shelf” platform with the potential to differentiate into any specialised lineage of immunomodulatory cells such as iPSC-derived Tregs and MSCs. This is scalability and cellular versatility which allows to produce specific immune effector subsets. Moreover, advanced genetic modifications of iPSCs using new and sophisticated genome-editing tools like CRISPR/Cas9 architectures allow for enhanced genetic engineering of safety and therapeutic profiles, and for cellular modifications that would diminish host immune rejection or boost anti-inflammatory activity both prior to and after clinical delivery of cells. In sum, the emerging advances in iPSC- and HSC-based strategies propel the cell-based immunotherapies of the future toward unprecedented opportunities in reprogramming the immune system and molecular engineering of immunopathologies [137, 138].

Reprogramming Efficiency: the discovery of effective reprogramming strategies using AI. By taking advantage of advanced systems biology and AI technologies, however, researchers have been able to evaluate many reprogramming methods and propose new ones with better efficiency and quality in iPSC generation [139]. AI techniques are helping to understand regulatory transitions and even to successfully monitor the progress of reprogramming.

#### Differentiation prediction

AI technologies have made possible the prediction of fate of differentiation in iPSCs and HSCs. For instance, the differentiating iPSCs to produce cardiac myocytes has been optimised thanks to improved reprogramming techniques to achieve cardiac myocyte strong generation without alterations to the genome [140]. Additionally, AI helps to improve the prediction of cell state landscapes and to estimate differentiation trajectories, which helps with the better stratification of patients and genetic phenotype modelling.

Clone Quality Assessment: The identification and assessment of iPSC clone quality is important in ensuring that they are effective and safe. Advances in AI, especially in image analysis, have made it possible to estimate the types of cell and their state precisely by morphological study. This capability is useful for assessing disease specific phenotypes and overall quality of iPSC clones [141].

#### Tumorigenicity risk detection

A major problem to the therapeutic application of iPSCs is the potential of iPSCs to be tumorigenic. AI technologies help to detect the genetic instability and assess the risk of developing tumour formation. Research has shown that some iPSC lines have been able to have pro-oncogenic states, demonstrable by technically demanding screening techniques such as in vitro cartilage tissue engineering, with a new function for preclinical assessment [142]. Likewise, image analysis by AI provides tools for identifying abnormal behaviour of cells that could indicate an elevation in their tumour-forming ability.

### Digital twins

In the Context of Autoimmunity Digital twins are patient-specific in-silico models that mimic biological processes and predict reactions to different treatments. These models are extremely useful for understanding the complex pathogenesis of autoimmune diseases, which involve complex immune responses and many factors, including genetic or environmental factors. It is important to clarify that digital twin modeling in the context of autoimmune stem cell therapy currently operates at the mathematical modeling and proof-of-concept computational stage. As of 2025, no randomized clinical trials have confirmed digitally-guided dosing, treatment scheduling, or patient selection for MSC, CAR-Treg, or iPSC-based therapies in autoimmune diseases. The studies reviewed here represent mathematical frameworks that simulate immune dynamics in silico and hold significant theoretical promise; however, translating them into clinical decision support tools requires prospective validation in adequately powered cohorts [143, 144].

The concept of a digital twin for autoimmunity is the production of a virtual representation of the phenotype of an individual patient’s disease. This is achieved by combining various sources of data, e.g. genetic data, molecular profiling, and clinical data into an integrated computational framework. These models can then simulate how the patient’s immune system could respond to various therapeutic interventions to enable the optimization of treatment protocols to the individual’s own unique biological characteristics. Mathematical modelling is an integral part of digital twins. Several models with different mathematical methods have been applied to describe some of the immune response relevant to autoimmune diseases, including ordinary differential equations and probabilistic models [145]. These models are an attempt to get mechanistic understanding on disease processes and predict the results based on hypothetical interventions. AI-driven therapeutic approaches are leading to the development of personalized medicine of autoimmune disorders. AI can utilize large patient datasets to make treatment more customized and improve the reliability of diagnosis, as well as discovering biomarkers [11]. Additionally, digital twins can help in the development of new therapeutic agents by allowing researchers to simulate the drug’s effects before the clinical trials [145]. This can greatly help reduce the time and resources required to develop effective treatments and ultimately benefit patients with autoimmune diseases by making more effective and safer treatment options available.

Though focused on MSCs, CAR-Tregs, and iPSCs, the predictive models and design rules built using AI lay groundwork seen later. These concepts resurface in targeted therapies examined closely by Sect.  4. Manufacturing standards following good practices, outlined in Sect.  6, rely implicitly on these earlier mechanisms. Logic developed here subtly shapes how scalable production takes shape downstream [116, 120].

## Disease-specific applications

### Systemic lupus erythematosus

Recent developments at the junction of AI and stem cell immunology have demonstrated the potential for the improvement of the treatment and management to SLE, particularly in the field of B cell receptor (BCR) and T cell receptor (TCR) repertoire modelling, CAR-T therapies, and relapse prediction.

#### BCR/TCR repertoire modelling

AI-based techniques have been very useful in enhancing the process of analysis of BCR and TCR repertoires in SLE patients. For example, phenotyping of molecular aspects of T-cell clones, such as CDR3 diversity and clonal composition, can be done in detail using high-throughput sequence technologies. These analyses can allow researchers to test the immune system in health and disease. Single-cell sequencing provides even more information through high-throughput study of individual TCR/BCR clonotypes to provide unique features of SLE that are distinct from healthy controls [139, 146–148].

The analysis data generated by TCR/BCR data with the help of an AI system has not only diagnostic relevance but also is directly relevant to therapy by guiding the choice of target antigen for future CAR-Treg designs specific to SLE pathobiology. As illustrated by the Müller et al. series [147, 149], for instance, identification of dominant pathogenic clones, such as CD19 + CD11c + age-associated B cells in a patient’s scRNA-seq-derived immune atlas could prioritize CD19 directed CAR-T as the therapeutic modality of choice. Further development of adaptive treatment scheduling, through the use of AI driven relapse prediction models that incorporate genomics, autoantibody dynamics and clinical trajectory further enables an optimised window for CAR-T infusion based on disease activity predictions [12, 150].

#### CAR-T therapies

Originally developed and optimized for use with cancer treatment, CAR-T cell therapies are now being studied for use for autoimmune diseases such as SLE. AI developments do help in devising an optimal design for CAR’s by modelling the immune response and predicting interactions of CAR with their antigen targets. Specifically, AI can be used to improve the effectiveness of therapies by predicting and designing antibody sequences that can improve the specificity and potency of these therapies [70].

#### Relapse prediction

AI is of great importance in predicting the relapses of SLE, which is the key for personalised treatment strategies. By combining a variety of multimodal data sources (genomics, proteomics, patient health data, etc.), machine learning models can be trained to identify high-risk patients and predict disease activity. For example, these models can use genetic information and clinical profiles to predict episodes of relapse to enable pre-emptive interventions. The AI application in here improves the precision and personalization of clinical treatments bringing about better patient management outcomes [12, 150].

### Multiple sclerosis

AI is increasingly being investigated as a tool to improve the interpretation of single-cell and spatial datasets related to the central nervous system (CNS) are interpreted, and will enable a better understanding and treatment of MS. AI’s data analysis functions are making waves in terms of comprehending the complex nature of datasets and the generation of stem cell therapies that induce tolerance.

#### Interpreting CNS single-cell and spatial datasets in MS

AI makes use of machine learning (ML) and deep learning (DL) algorithms for improving the analysis of complex datasets from the human CNS. These techniques allow one to extract detailed features from different imaging techniques such as retinal optical coherence tomography (OCT) and MRI, vital for detecting and tracking MS-related abnormalities. For example, OCT analysis is improved by AI to enhance the accuracy of classifying the condition by detecting subtle changes in retinal layers that indicate MS-related pathology [151, 152]. AI’s interpretative abilities are also working for models of unsupervised machine learning, which classify subtypes of MS based on brain MRI data. By uncovering groups with alike pathological characteristics, AI helps forecast disease development and group patients for tailored therapeutic treatments [153]. Furthermore, multimodal datasets have been used by deep learning studies to forecast the severity of MS, demonstrating the power AI has to determine a disease with greater accuracy, early diagnosis [154].

MS lesions have been analyzed for immune cells, which identified immune sites (niches) in the perivascular spaces flanking multiple lesions dominated by exhausted CD8 + T cells and inflammatory microglia that might be impervious to systemically administered tolerogenic stem cell therapies [155]. This spatial information allows for precision targeting - targeting stem cell derived immunomodulatory products to specific CNS compartments, or targeting patients for whom lesion microenvironments are more immune regulation friendly [153, 154]. AI tools for MS subtyping based on MRI and spatial immune data could be used to identify the patient population most likely to benefit from immune reconstitution strategies, including the setting of MS with active inflammatory lesions and not progressive disease with atrophy-dominated disease [153]. AI MS subtyping technologies based on MRI and spatial immune information may help identify the patient population most likely to benefit from immune reconstitution strategies, including in MS patients with active inflammatory lesions and not progressive disease with atrophy-dominated disease [154].

#### Supporting tolerance-inducing stem-cell therapies

AI has a role in stem cell therapy in terms of perfecting the process of inducing immune tolerance, which is a very important part of treating auto-immune diseases such as MS. The combination of AI and research into stem cells allows for more accurate and personalized treatment options by enabling the construction of tolerance-inducing treatment protocols and evaluating them. AI models can be used to simulate complex interactions between the immune system, and this can help to predict how patients may respond to stem cell therapies and optimise treatment plans [156].

### Type 1 diabetes

AI is becoming a more important factor in addressing complex healthcare problems, especially in the treatment and management of T1D. As an example of AI contribution to autoantigen discovery, T-cell receptor (TCR) specificity prediction and strategy involving induced pluripotent stem cells (iPSCs) in T1D.

#### Autoantigen discovery

AI has revolutionized the autoimmune discovery of autoantigens which play a key role in the understanding of autoimmune diseases such as T1D. Techniques like Phage Immunoprecipitation Sequencing (PhIP-seq) provide autoantigen identification in a proteome wide unbiased manner and models based on AI and machine learning improve this process to allow scalable analysis of large comprehensive datasets [157, 158]. AI helps in the detection of new autoantigens that can be used in the understanding of the mechanism of the disease and its possibility target therapy [159].

#### TCR Specificity Prediction

Predicting TCR specificity is of importance for understanding immune responses in T1D. AI models, such as RACER-m and STAPLER, reduce to structural features, that is, machine learning algorithms that are used to predict TCR recognition of peptide-MHC (pMHC) complexes with high sensitivity and specificity. These models address the limitations in sparse training data and economize our modeling of correctly modeling of TCR interaction These models address the limitations of sparse training data and improve our ability to accurately model TCR interactions [160, 161].

#### iPSC Derived Beta Cell Transplantation

More specifically, in the case of T1D iPSCs are showing promise as a source for generating B-cells, as they can provide an essentially unlimited source for cell replacement treatments. AI helps in optimizing the process of differentiation and transplanting iPSCs into functional insulin-producing b-cells. Various researches demonstrate that iPSCs can be developed into b-cells that secrete insulin and efficiently control blood glucose level [162, 163]. However, issues such as immune rejection and how to make the cells maintain their functionality post-transplantation still pose a problem. AI can contribute to resolving these problems by optimizing the protocols and determining the prediction in order to improve the effectiveness of such therapies [164].

### Rheumatoid arthritis & inflammatory bowel disease

AI is increasingly being explored to improve the management of autoimmune diseases such as RA and IBD through the mapping of the synovial and gut environment and the categorization of patients for specific treatments such as mesenchymal stem cell (MSC) or regulatory T cell (Treg) therapy. In the realm of IBD, AI is gaining a substantial amount of use, especially in its use to explore the complex interactions of genetic, microbial and environmental factors contributing to the Development of IBD [165]. The application of AI and machine learning methods is therefore useful to tease apart these complex interactions and enable researchers to identify biomarkers and potential therapeutic targets that can be used for better patient management and targeted therapies [166]. AI is used to analyze multiomics data - like genomics, transcriptomics, proteomics in order to understand pathogenesis of IBD in favour of creating targeted therapies like MSC therapy [165, 167]. The use of AI in the diagnostics process increases the stratification of patients who could benefit from innovative therapies. AI models have been developed to detect universal IBD-associated bacterial species, which can serve as non-invasive diagnostic tools. This method not only helps in the diagnosis of IBD but also assists in monitoring the disease course and predicting response to treatment, which in turn can be used to target treatment with specific therapies, such as Treg therapy [168, 169]. Furthermore, in the context of RA, there are applications of AI in synovial niche mapping that are based on multi-omics data, to comprehend immune responses and pathways of the disease. This approach can be extensive and integrated which can find out patients who can benefit from targeted therapies and this may lead to personalization of treatment plan and improves therapeutic outcomes [170]. Overall, the ability of AI to handle and interpret large data pairs and effectively process them is paving the way to innovative methods of stratifying patients and aiming at disease treatment via targeted therapy approaches in the management of complex diseases, such as RA and IBD [171, 172].

For example, in the mouse model of RA, innovatively innovated mapping through AI has identified that there are at least three different subpopulations of stromal fibroblasts — CD90 + sublining fibroblasts, NOTCH3 + perivascular fibroblasts, and Thy1-negative lining fibroblasts — with distinct functions in joint destruction and immune exclusion [173, 174]. It has direct implications for the design of MSC therapy: researchers see less promise in systemic MSC infusion in Thy1-negative synovial biopsies compared to CD90 + sublining enrichment [55, 173, 174], and the data suggest that CD90 + sublining enrichment would benefit from intra-articular delivery of MSCs based on AI predictions regarding homing receptor compatibility. The ability of AI multi-omics integration to classify patients into endotypes was effectively used to identify patients with high intestinal regulatory T cell deficit — who represent the most biologically rational and suitable cohorts for cell transfer based trials of intestinal diseases such as IBD — turning AI-based endotype classification into an end-to-end patient selection strategy for cell transfer trials [168, 175, 176].

Psoriasis and vitiligo are examples of dermatologic autoimmunity that is still mostly investigational with AI directed stem cell therapy. But single cell atlas studies in psoriatic skin has identified the CD8 + tissue-resident memory T cell (TRM) as the main pathogenic population suggesting that antigen-specific Treg Therapy targeting melanocytes and keratinocytes autoantigens might be a biologically sound therapeutic direction [177, 178]. Non-responsiveness to IL-17 or JAK inhibition may be predicted from before treatment by single-cell classifiers derived from pre-treatment biopsy skin transcriptomes, and therefore the individuals who do not respond are potential candidates for future interventional trials using Tregs [179, 180].

### Dermatologic autoimmunity

AI, and in particular machine learning (ML), plays an important role in the evaluation of single-cell atlases of skin diseases such as psoriasis and vitiligo in order to develop classifiers to predict responses to therapy. In dermatology, AI helps us understand the complicated disease mechanisms and help in designing personalized treatment mechanisms. AI systems use the information obtained from single-cell transcriptome sequencing to provide detailed information about cellularity in diseased skin areas of conditions such as psoriasis and vitiligo. These analyses aid in the identification of specific immune cell populations and the functions of these cell populations in the disease process. For example, increase in the number of CD8 + T cells, natural killer cells and various types of dendrite cells have been detected in the skin tissues of patients with such conditions [178]. This information is important to understand the pathophysiology of these autoimmune diseases, which are characterized by abnormal immune responses [177]. A branch of AI, machine learning algorithms, are important in analyzing skin images to identify and classify skin lesions, helping with skin lesion diagnosis and severity evaluation for psoriasis [180, 181]. By training models with large datasets of dermatological pictures, AI can learn to gain high levels of correctness in determining to recognize paternities related to various disease states, which is important in predicting treatment outcomes of the same. These algorithms are trained to-recognize and separate lesions, determine the severity of the disease and even predict the potential complications the disease may have [182]. As far as therapy response prediction is concerned, single-cell data response prediction is used by AI to find biomarkers and to determine how well the patient will respond to certain treatments. This approach opens up the possibility to develop classifiers that can predict the therapy success on the basis of cell and molecular features. The identification of molecular pathways and immuno mechanisms involved in these skin diseases have led to the utilization of targeted therapies, such as immunomodulating agents and IL-17 inhibitors which exhibit different levels of efficacy [183, 184]. Furthermore, AI has made it possible to integrate data from different sources such as genomics studies, clinical trials, and more to create comprehensive models that lead to even better predictive powers and insights into possible new therapeutic targets. Despite the promising developments, challenges like the question of model validation, the diversity of training data and their incorporation into workflows in a clinical setting remain to be addressed for successful implementation in clinical practice [181].

## Translational case studies

### CD19 CAR-T in autoimmunity

CD19 CAR-T therapy is one of the promising approaches to treatment autoimmune disorders, including autoimmune SLE, idiopathic inflammatory myositis. Originally developed to treat haematological cancers, such therapy has been adapted to treat stubborn autoimmune diseases, and there are promising results.

#### Clinical trials in lupus and myositis

There have been recent case series and clinical trials that have investigated the efficacy of CD19 CAR-T therapy for autoimmune diseases. A study done on 15 patients with severe SLE, idiopathic inflammatory myositis, and systemic sclerosis showed efficacy of a single infusion of CAR-T cells expressed against CD19 in inducing remission. Specifically, all the patients with SLE met the DORIS remission criteria and those with myositis showed major clinical responses based on the ACR-EULAR criteria. One of the most important treatment effects was B cell aplasia, which lasted an average of 112 days, making possible a long period without the need of medications [149]. In a separate small case series looking at treating resistant SLE, 5 patients that did not respond to standard treatments were treated with CAR-T infusions. This led to the ridding of autoreactive B cells and improvements in clinical conditions and normalization of lab markers. The therapy was effective and well-tolerated by the patients, and maintained remission even following the reappearance of B cells [185].

#### Adverse events and management

CRS is a common CAR-T treatment side effect that is usually experienced in a light form with adverse effects that can be managed. In one case, there was a patient who developed grade 2 CRS and grade 1 neurotoxicity, both of which were successfully treated with standard treatments [149, 185]. Despite these side effects, the safety profile of CD19 CAR-T in the treatment of autoimmunity remains positive, given its beneficial effects in the therapeutic field.

#### Potential use of AI in optimizing therapy

AI plays a crucial role in the improvement of the processes associated with patient selection, monitoring and management for CAR T therapy. In medical fields such as rheumatology and other disciplines, AI algorithms possess the ability to predict how patients will respond, identify patients who may benefit the most from treatment, and tailor treatment plans specifically to individuals [186]. By using machine learning methods, large datasets may be analyzed to find patterns and markers predictive of successful outcomes to enhance CAR-T therapy. AI technologies are able to track the progress of people subjected to therapy, making it possible to detect relapses or adverse effects such as CRS at an early stage. With the help of real-time information, collected by electronic health records and wearable devices, AI can generate proactive management plans, thus ensuring the intervention is carried out on time and the patient safety is ensured [186]. Initial clinical trials and case studies of CD19 CAR-T therapy in autoimmune diseases such as lupus and myositis show great promise for getting people into remission from diseases where conventional treatments have been unsuccessful. As this therapy gains momentum, it is possible that the use of AI could help to further improve patient outcomes through an improved personalization of treatments and monitoring of safety.

### Artificial intelligence-optimized MSC potency

The use of AI-based technologies in conjunction with cell biology is changing the landscape of mesenchymal stromal cell (MSC) characterization and functional optimisation. Two essential elements that underpin this progress were the use of metabolomics and image analysis coupled to an AI system in order to identify senescence markers and the use of an AI-based predictive modelling approach that proposes dynamic and adaptable strategies of intervention to reduce and preserve the MSC immunomodulatory capacity during scalable production.

#### Morphological analysis and senescence detection

AI-driven advanced models made by deep learning technologies can accurately detect senescence in MSC cultures by morphological changes. For example, a morphine-based cascade R-CNN system has been developed to recognise senescent MSCs. This system enables automatic identification and evaluation of individual cells in multi-cellular images that representative of morphology in connection with other signs of senescence. The deep learning model is also effective in the detection of both chronic and acute MSC senescence, which provides a cost-effective and labour-saving approach to screen MSC culture conditions and anti-aging treatments [116].

#### Senescence biomarkers & metabolomics

Among the AI tools that have been developed to analyze untargeted metabolomics data and brightfield microscopy images are so-called machine learning classifiers, random forest algorithms, and deep convolutional neural networks (CNNs), which allow the identification of complex, high-dimensional patterns without destroying cells. Metabolic hallmarks like changes in NAD+/NADH ratio and lipidomic remodeling are indicators for senescence of MSCs and indicative for intracellular redox imbalances and membrane biogenesis perturbations. Brightfield deep-learning models can reliably predict senescence-associated outputs, such as beta-galactosidase activity and cell cycle arrest markers for studying cell viability and heterogeneity profiling without labels [187]. At the same time, machine learning of an untargeted metabolomics profile identifies more subtle, non-intuitive biomarker clusters associated with senescence, which are more powerful than typical univariate analyses. Random forest algorithms have specifically shown good performance in feature selection, allowing identification of key metabolites to distinguish cellular aging states, thus revealing new metabolic signatures that can be monitored in real time during the expansion of MSCs.

#### Intervention strategies

Following the identification of biomarkers, AI-powered predictive models and systems based on reinforcement learning algorithms conduct in silico experiments and simulations of metabolic fluxes and cellstate changes in order to optimize tailored interventions. These computational frameworks combine multi-omic and imaging information to predict the skew of senescence and MSC immunomodulatory function in response to various culture perturbations such as media composition, temporal nutrient perturbations, and small molecule perturbations effecting MSC function. As the AI model iterates over parameter space, it outputs the optimal formulations and windows of time that will halt the replicative senescence progression and rejuvenate therapeutic potency – dynamically. Reinforcement learning (RL) algorithms continuously optimize their strategy by using the expected outcomes of the simulated strategy, which allows for closed-loop optimization of the culture protocol in large-scale biomanufacturing. This paradigm is crucial for scaling up and ensuring product consistency in current applications facing culture-induced heterogeneity and phenotypic drift issues [187, 188]. The metabolic flux prediction applied to the media design along with AI-driven media engineering allows tailoring MSC manufacturing workflows to maintain cell functionality and potency, thereby enhancing the clinical efficacy of MSC-based therapies.

#### Translational implications

The integration of the AI in predicting optimizing MSCs has a significant effect on clinical applications. By understanding the factors and conditions that can affect the aging and function of MSCs, AI technologies have helped in standardizing the production of MSCs with improved therapeutic potential. The real-time analysis offered by morphology-based AI systems allows guiding the modification of the culture conditions and interventions to extend the functional life of MSCs. Furthermore, metabolomics integrated with AI technology has made it feasible to find metabolic changes related to cellular aging, providing a target for pharmacological intervention for the potential rejuvenation of senescent cell [117, 189].

### Artificial intelligence-driven patient sratification and clinical outcomes

AI patient stratification in clinical outcomes is a method of applying AI technology for predicting patient outcomes in the case of autoimmune disease management. AI patient stratification systems create predictions of stem cell potency in terms of stem cell different ability due to the efficient and accurate analysis of imaging and multiple omics data. This information also produces critical quality attributes (CQA) during the monitoring of production, for example, cellular morphology and cellular growth rates will be performed in real-time, ensuring products can be reproducible and scalable for clinical manufacturing [190, 191]. Also, systems biology with the application of AI (SysBioAI) allows researchers to relate the specifications of a stem cell product to how a patient reacts to treatment. This is accomplished by facilitating the translation of stem cell products into clinical use, as well as identifying predictive markers prior to clinical use [192].

Moreover, the identification of patient outcomes will continue to improve more effectively through AI guided machine learning algorithms. Such as through auto metamorphic disease processes (e.g. RA and SLE), where multiple parameters (clinical and non-clinical data) are evaluated using other systems and data sources to help create profiles for the different patients with RA and SLE. In autoimmune rheumatic diseases, the prediction of pathogenic variants in deep learning has augmented the potential to make better therapeutic decisions by using patient-specific genomic signatures to guide those decisions [11, 12, 193]. In RA, for example, the incorporation of imaging and molecular data into machine learning prediction algorithms or models has resulted in developed personalized biologic therapies; while in SLE, AI has enabled accurate predictions of disease activity resulting in fewer clinical flare-ups [194].

These AI-powered platforms will also support the development and use of tools that will improve the efficiency of drug discovery process, improve how drugs are delivered, and how they differentiate prior to being administered. By simulating how stem cells will respond in various situations and anticipating clinical trajectories, AI will allow for the implementation of preventive measures by modifying treatment plans to make stem cell therapies safer, more individualized and more effective for the treatment of autoimmune illnesses [194, 195].

## Artificial intelligence in GMP manufacturing & regulation

### Automated GMP systems

Incorporating AI in GMP frameworks, especially for stem cell treatments, these technologies provide the potential and functionality to increase manufacturing efficiency, improve product quality, and help to manage treatments in real-time. In the centre of this integration lie closed-system bioreactors and real-time monitoring.

#### Real-time monitoring

Real-time monitoring is essential to ensure that the GMP system processes are within set parameters. AI and machine learning allow for real-time data analysis and helps to quickly respond to any potential issues. For instance, combining AI with sensors and imaging technologies can provide real-time feedback information on the cell morphology and growth levels. Advanced natural language processing (NLP) methods enable the extraction and investigation of data for stem cell therapy from electronic health records (EHRs) to help better adhere to the process and control [196, 197].

#### Data management and predictive analytics

AI not only assists in adjusting processes in real-time, but also assists in improving processes at the long-term level by means of data management and predictive analytics. The combination of machine learning strategies and historical data in AI models can predict future trends and results in stem cells production, aiding the improvement of SOPs (Standard Operating Procedures) and maintaining standard product quality [198, 199].

#### Ethical and regulatory considerations

As AI systems get entrenched in GMP processes, transparency is key to addressing the decision-making process around AI. Explainable AI (XAI) which ensures that AI systems can make understanding and interpretable decisions that are in line with the regulatory requirements and ethical. Ethical issues like data privacy, and bias in AI models need to be resolved to be compliant with GMP standards and preserve the trust of the public [200]. By utilizing the capabilities of AI, GMP systems for stem cell therapies can make deployments far more precise and reliable ultimately improving the outcome for patients and the field of regenerative medicine.

### Quality control & potency prediction

AI-based quality control (QC) is pivotal in the effectiveness and security of MSCs and induced pluripotent stem cells (iPSCs) by developing imaging, omics, and reinforcement learning to find the best process. Each of these AI technologies is unexplored in providing benefits to the QC process.

#### Imaging and machine learning

AI has significantly improved imaging-based analysis. By using a combination of high-throughput imaging and AI, cellular morphology can be accurately analyzed to predict cell types and states using basic microscopic images [201]. Machine learning models are known to be able to classify the iPSC-derived cells and evaluate the differentiation stages, which facilitates the standardization and efficiency of iPSC-based products [202].

#### Omics technologies

The combination of data from omics technologies can provide comprehensive information on cellular function and genetic stability that is important for ensuring stem cell safety AI tools can analyze genomic, transcriptomic and metabolomic data to forecast phenotypes and make sure that cells retain desired traits when they are being differentiated and proliferated. This helps to knows the efficiency and safety of stem cells for many therapeutic purposes [203].

### Regulatory issues

Regulatory guidelines for AI in Advanced Therapy Medicinal Products (ATMPs) are primarily concerned with providing transparency, interpretability, validating the dataset and ensuring that the model creates an audit trail. Without these components, progress in AIpowered medical advances could falter due to compromised safety or weakened confidence among users.

#### Transparency

Transparent AI systems are important in healthcare because they allow healthcare stakeholders to understand how AI systems make decisions and determine whether these decisions are fair and accurate. Functional transparency means an understanding of the inner workings of AI systems and the ability to effectively communicate such workings to the users. This corresponds with the need for clear as well as accessible explanations of AI processes to create trust between clinicians and patients [204].

#### Interpretability

Interpretability means making the decisions made by AI understandable to stakeholders, who are human beings. Techniques like Explainable AI (XAI) therefore try to solve the “black-box” side of AI with explanations of the decisions it makes. This can involve various methods, such as model-based, attribution-based or example-based explanations, and can be implemented as part of the AI model (global) or as separate modules (local) for a specific decision [205, 206].

#### Validation datasets

The use of strong and right validation datasets is basic to the world of AI model evaluation. These datasets are useful in assessing the performance, accuracy and generalisability of the AI algorithms. They are important in establishing the reliability and credibility of AI models in the clinical setting. Particularly in AI-based medical devices, this entails gaining insights about the performance of models with various clinical information and validating that these models are applied across different demographics and spectrum of diseases [207].

#### Model audit trails

Audit trails in AI systems are important in the paradigm of adherence to regulations and accountability. They record the development, training and deployment of models and how they changed and developed over time. This documentation means that AI systems can be scrutinised and analysed retrospectively and that is important in order to address any issues which arise during their use. Audit trails - Helping maintain the integrity of AI models, audit trails help to track retraced data provenance, modifications to data and decision-making processes [208]. Overall, these regulatory expectations make sure that the utilized AI in ATMPs is put into practice safely and effectively, with a high level of trust, and acceptance, from healthcare professionals as well as their patients. By promoting transparency, interpretability, validation and accountability, regulators are seeking to reduce the risks and amplify the benefits of using AI technologies in complex medical applications.

## Ethics, equity & reproducibility

AI has significantly advanced in stem cell immunology raises many issues related to ethics, including issues of bias, reproducibility, genomic privacy and interpretability.

### Bias (ancestry, sex)

AI solutions can systematically perpetuate bias, particularly when the source is biased training data in that the training dataset lacks diversity by way of representation of diverse populations. Such biases in AI can lead to unequal healthcare outcomes, because of the injustices that might arise from not considering the differences based on ancestry or even sex. To correct for these types of biases, it is important to ensure that training datasets are comprehensive and representative and are inclusive of the diverse genetic and demographic backgrounds of potential patients [209].

### Reproducibility

Reproducibility of AI models in biomedical research continues to represent a fundamental challenge. Models are often trained and validated on single-institutional datasets, which may not generalize to independent cohorts owing to variations in sequencing platforms, patient demographics, or tissue processing protocols. To ensure reproducibility necessitates clear methodological reporting, including complete disclosure of preprocessing pipelines, hyperparameters, and training-validation splits, along with open code and data sharing wherever ethically possible [210]. Standardized reporting frameworks for AI in biomedicine such as TRIPOD-AI and CLAIM provide template structures improving the transparency and reproducibility of published models, and their adoption should be encouraged in the stem cell immunotherapy field [211, 212].

### Genomic privacy

The management of genomic information raises privacy issues because of the sensitive nature of genetic information. AI systems in stem cell immunology have to comply with stringent data privacy laws and ethics to ensure individual privacy. Implementing robust data encryption and anonymization methods and ensuring that privacy laws such as GDPR are followed can help to protect people’s genetic information from silent access or misuse [213].

### Interpretability

It is crucial to have a level of interpretability for the AI models that are being developed for clinical use to ensure that healthcare professionals can understand and trust AI-based decisions. Models that are “black boxes” are hard to explain, which is often a setback in acceptance of them in healthcare practices. Explorable AI (XAI) methods like December trees, feature importance and visualizations can be used to provide transparency in model making that would assist healthcare providers in understanding the decision-making processes [214, 215].

## Current limitations and Future perspective

Despite the remarkable advances in AI-driven stem cell immunology, several limitations continue to reach clinical translation. One major challenge is the “black-box” nature of many machine learning and deep learning models, where predictions are generated without transparent biological explanations. Although highly accurate models may be useful for prediction, lack of interpretability can limit regulatory approval, and mechanistic understanding. Consequently, Explainable AI (XAI) approaches are increasingly being developed to improve transparency and trustworthiness. Another significant limitation is the scarcity and heterogeneity of autoimmune disease datasets. Autoimmune disorders are characterized by diverse phenotypes, and relatively small patient populations. Under such conditions, complex AI models are susceptible to overfitting, results in models that perform well on training datasets but fail to generalize to independent cohorts. Robust external validation, multicenter collaborations, and standardized data collection frameworks are therefore essential.

The potential of AI in healthcare and research is enormous with the advancements in multimodal foundation models, generative AI, spatial-aware models, digital twins, and combination of AI and laboratory processes. These innovations will surely change the methods of old and increase precision, efficiency and personalization related to therapies optimization. By bringing a number of different data forms, ML models can improve the learning and predictive powers across domains. Specifically, their application in medical image interpretation has shown that although they could enhance the diagnostic reasoning process, they possessed a strong dependence on textual inputs that could be a major contributor to performance when presented with erroneous text information. Future bids would be working on improving these models so that the text and visual inputs can be balanced, making medical diagnostics reliable and accurate [216]. Additionally, generative AI is having a momentous impact on antigen discovery by optimizing antibody design and optimization, based on superior data analysis. This technology enables faster generation and accurate prediction of immune response and enhance therapeutic efficacy which is vital in personalized vaccine development. The combination of machine learning and AI in antigen design is expected to suit the existing issues in vaccine development including scarcity of data and the model’s ability to be interpreted better [70]. Apart from antigen designing, there is digital twins, which offer real-time, data-driven simulations of biological systems to support personalized medicine. These digital models can replicate patient-specific conditions, enhance treatment plans and forecasting disease progression. The fusion of AI with digital twins is crucial for advancing healthcare outcomes and holds potential in areas like cardiovascular care and smart farming, where real-time comprehensive data analysis can lead to better decision-making and increased operational efficiency [144, 217]. Another innovation on Closed-Loop AI-Lab Integration for Therapy Optimization Integration of AI and closed-loop is set on the way in revolutionizing therapeutic management in healthcare. These systems that adaptively respond to the inputs received from the patient and adjust the therapeutic measures, accordingly, are making the way for intelligent and automated healthcare solutions. For chronic diseases such as diabetes, closed-loop systems have shown great potential for glycemic control and an improved quality of life. The future of therapy optimization will be strongly based on such systems that integrate real-time data and feedback with AI-driven decision-making capabilities to offer personalised, efficient and adaptive treatment solutions [218, 219].

### Critical challenges in artificial intelligence-guided stem cell immunotherapy

A lack of larger scale clinical validation. Most of these AI models that are applied to the immunotherapy of stem cells have been trained and tested in small, single-center collections or retrospective cohorts, or in a purely in silico setting. AI models for prediction of MSC donor potency and CAR-T toxicity have shown encouraging results in retrospective studies but remain to be tested in prospective studies with adequate sample size and multicentric validation [74, 117, 118]. The most critical unmet need in the field is this gap between the ‘proof of concept’ in the computer and clinical proof. Future clinical trials with AI decision support tools should include the decision threshold and test set in the clinical trial protocol, as is done for companion diagnostics, and specification of the AI model and its validation set [207].

### Dataset bias and lack of diversity

To create AI-driven models, most single-cell or multi-omics training data sets in the public domain are from Europeans and North Americans, and South Asian, East Asian, African and Latin American ancestries are substantially under-represented [220]. One ancestral bias that is especially relevant to autoimmune disorders is the many different clinical phenotypes, HLA associations, and responses to therapy found among ethnic groups, many of which, such as SLE, RA, and IBD, happen to be autoimmune disorders. When AIs are used in the global clinical setting, they could expand or reinforce health inequities when trained on non-diverse data sets. For these reasons, a number of corrective measures have been proposed, such as the development of federated learning frameworks which allow the training of models by training on geographically distributed cohorts without centralizing patient data, and algorithmic fairness auditing that assesses the performance of the model stratified by ancestry, sex, and age [221].

### Reproducibility and batch effect challenges

Technical batch effects, such as those due to different sequencing platforms, library preparation protocols, cell isolation processes, and laboratory conditions, have been known to affect high-dimensional omics data used to train AI models in autoimmunity studies [56, 222]. If there is not a strict batch correction imposed, for example by tools like harmony, combat or scVI, AI models may learn spurious technical signals instead of real biological variation and generate success within their own cohort, but not outside of it. To develop reproducible AI models in stem cell immunology, there are numerous prerequisites that include standardized preprocessing pipelines, open code repositories, and community benchmarking initiatives as vital prerequisites [56, 223].

### Explainability and the ‘Black Box’ problem

There are numerous high-performing AI systems in stem cell immunology, such as the deep learning-based senescence detectors, CNNs for iPSC quality classifiers, and graph neural networks for prediction of the state of immune cells, that are “black boxes” with an unknown decision criterion, inaccessible to clinicians and regulators [205, 206, 224]. This lack of explainability makes it difficult to adopt into real clinical scenarios and trust by any institution or regulatory body because clinicians do not have a means to determine if the model is reasoning based on biologically meaningful features or exploiting features in the dataset. Biomedical models are being used with XAI algorithms like Shapley Additive exPlanations (SHAP), Local Interpretable Model-agnostic Explanations (LIME), and attention visualization in transformer networks to better understand which input features are most important for each prediction [205, 206, 225, 226]. Whereas for AI tools employed to make decisions in a GMP or inform clinical patient allocation, explainability is a regulatory obligation [224].

### Regulatory framework for artificial intelligence in advanced therapy medicinal products (ATMPs)

AI frameworks are being actively designed by regulatory bodies to integrate into the clinical development of Advanced Therapy Medicinal Products (ATMPs), such as stem cell-based therapy. The US Food and Drug Administration (FDA) has issued guidance on Software as a Medical Device (SaMD) and AI/ML-based software in medical devices, emphasizing the need for predetermined change control plans, performance monitoring, and audit trail documentation [227]. The European Medicines Agency (EMA) also calls for AI tools used within the manufacture of ATMPs and clinical medical decisions to be both transparent and validated, with demonstrated performance in demographic subgroups and robustness against input variations. The models should be documented and traceable as required for any other PATs (Process Analytical Technology), and should be subject to the same version control, re-validation triggers, and continuous performance monitoring after deployment as any other PAT system [228].

### Data privacy and federated learning solutions

AI models that are useful for stem cell immunotherapy depend on availability of extensive, well annotated patient data, including genomics, single-cell transcriptomics, clinical information and therapeutic responses, which must adhere to stringent privacy regulations like GDPR in Europe and HIPAA in the United States [221]. It is generally not legally or ethically feasible to have so much of this important data reside centrally across institutions. Federated learning, where an AI model is trained locally at each contributing site, with only updates to a model’s weights being shared, provides a principled solution that keeps patient data private and allows for multi-institutional training and validation [221]. The federated approach to multi-center cohorts for scRNA-seq models or GMP process models could significantly improve the generalizability of these field models while maintaining patient anonymity.

## Conclusion

The process of AI in stem cell immunology has significantly enhanced the research and clinical applications of treatment for autoimmune diseases. AI has helped in the analysis and synthesis of complex datasets and thereby enhanced knowledge of MSCs and their immunomodulatory roles. Utilizing AI researchers can predict and improve therapeutic outcomes; tailor therapies according to personal genetic profiles and identify potential biomarkers for the better management of diseases. AI-driven models enable precise modulation of stem cell function (Fig. 1). Notably the strategy of the use of mesenchymal stem cell-derived exosomes (MSC-EXOs) has emerged as a promising strategy because these exosomes contain bioactive substances that can effectively modulate the immune response without requiring direct interaction between the two cells. To further increase AI’s effects in stem cell immunology is also a number of recommendations. First, interdisciplinary collaborations should be encouraged to combine AI technologies with classical immunological approaches serving as a comprehensive approach to the treatment of autoimmune diseases. Secondly, the research should focus on improving AI algorithms to minimize biases and improve the accuracy and reliability of predictive models. Additionally, creating standardized protocols to address data management and privacy can help to solve problems that pertain to data quality and data security. Lastly, clinical trials should be done to study the long-term effects of AI-enhancer stem cell therapies to ensure their safety and efficacy for different patient populations.

## Data Availability

No datasets were generated or analysed during the current study.
